# Modelling the potential spatial distribution of mosquito species using three different techniques

**DOI:** 10.1186/s12942-015-0001-0

**Published:** 2015-02-27

**Authors:** Daniela Cianci, Nienke Hartemink, Adolfo Ibáñez-Justicia

**Affiliations:** Faculty of Veterinary Medicine, Utrecht University, Utrecht, The Netherlands; Centre for Monitoring of Vectors, Food and Consumer Product Safety Authority, Wageningen, The Netherlands

**Keywords:** Species distribution modelling, Non-linear discriminant analysis, Random forest, Generalised linear model, Indigenous mosquito species, Vector-borne diseases

## Abstract

**Background:**

Models for the spatial distribution of vector species are important tools in the assessment of the risk of establishment and subsequent spread of vector-borne diseases. The aims of this study are to define the environmental conditions suitable for several mosquito species through species distribution modelling techniques, and to compare the results produced with the different techniques.

**Methods:**

Three different modelling techniques, i.e., non-linear discriminant analysis, random forest and generalised linear model, were used to investigate the environmental suitability in the Netherlands for three indigenous mosquito species (*Culiseta annulata*, *Anopheles claviger* and *Ochlerotatus punctor*). Results obtained with the three statistical models were compared with regard to: (i) environmental suitability maps, (ii) environmental variables associated with occurrence, (iii) model evaluation.

**Results:**

The models indicated that precipitation, temperature and population density were associated with the occurrence of *Cs. annulata* and *An. claviger,* whereas land surface temperature and vegetation indices were associated with the presence of *Oc. punctor*. The maps produced with the three different modelling techniques showed consistent spatial patterns for each species, but differences in the ranges of the predictions. Non-linear discriminant analysis had lower predictions than other methods. The model with the best classification skills for all the species was the random forest model, with specificity values ranging from 0.89 to 0.91, and sensitivity values ranging from 0.64 to 0.95.

**Conclusions:**

We mapped the environmental suitability for three mosquito species with three different modelling techniques. For each species, the maps showed consistent spatial patterns, but the level of predicted environmental suitability differed; NLDA gave lower predicted probabilities of presence than the other two methods. The variables selected as important in the models were in agreement with the existing knowledge about these species. All model predictions had a satisfactory to excellent accuracy; best accuracy was obtained with random forest. The insights obtained can be used to gain more knowledge on vector and non-vector mosquito species. The output of this type of distribution modelling methods can, for example, be used as input for epidemiological models of vector-borne diseases.

## Background

Mosquitoes (Diptera:Culicidae) are known to be vectors of a large number of pathogens around the globe. Blood-feeding females of several mosquito species are involved in transmission of protozoa (e.g. *Plasmodium*), nematodes and viruses. Mosquitoes are considered as prime candidates for transmitting (re-)emerging vector-borne diseases in Europe [1].

Accurate information on the spatial distribution of mosquito species is essential for our understanding of the current risk of diseases transmitted by mosquitoes and for preparing for future threats [2]. For the modelling of the spatial distribution of species, several techniques exist [3,4], differing in assumptions and predictive performance. The general idea behind species distribution modelling is to identify relationships between known occurrence of a species (presence/absence) and environmental data (e.g. meteorological data, land use covers, remote sensing data) and to use these relationships to make predictions for all unsampled areas in the study region.

Here, we compare non-linear discriminant analysis (NLDA), random forest (RF) and generalised linear models (GLM), three techniques that have not been compared before, by applying them to a new dataset consisting of systematically collected data on three indigenous mosquito species in the Netherlands. The three mosquito species are *Culiseta annulata* (Schrank, 1776), *Anopheles claviger* (Meigen, 1804) and *Ochlerotatus punctor* (Kirby, 1837). For each of these species, the resulting environmental suitability maps and the most important environmental variables selected by the models are discussed and the techniques are compared in terms of model performance.

## Results

The environmental suitability for *Culiseta annulata*, *Anopheles claviger* and *Ochlerotatus punctor* was investigated in the Netherlands using three statistical models, i.e., NLDA, RF and GLM. Through these modelling techniques, occurrence data collected in 766 locations were linked to environmental factors.

**Figure 1:**
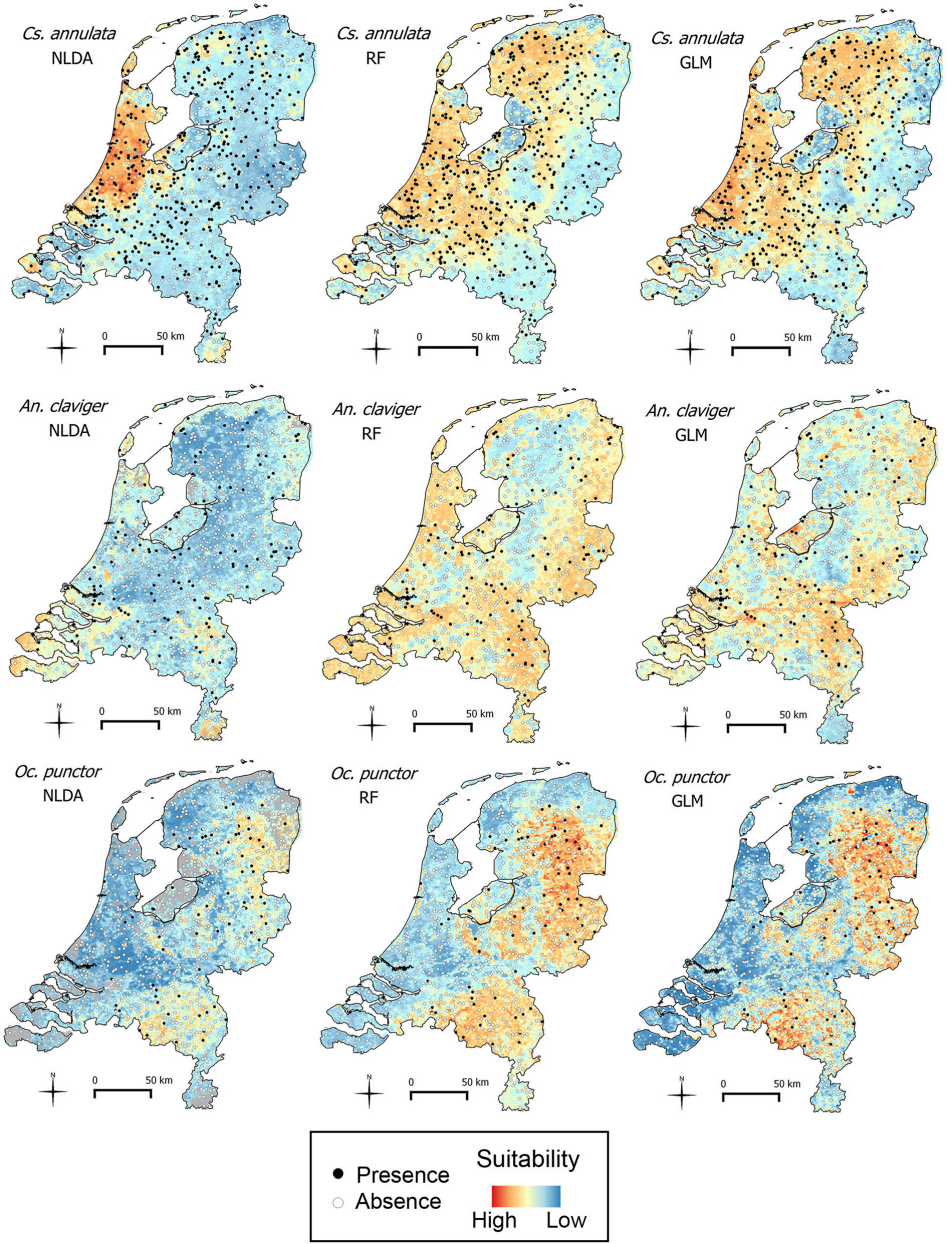
**Environmental suitability maps.** Environmental suitability maps for *Cs. annulata*, *An. claviger* and *Oc. punctor*, produced using non-linear discriminant analysis (NLDA), random forest (RF) and generalised linear model (GLM). Black dots indicate that the species was captured on the sampled locations and white dots indicate that the species was not captured. Environmental suitability is depicted using a gradient fill: blue indicates low environmental suitability, red indicates high suitability. NLDA and GLM bootstrapping was based on 150 presence points and 150 absence points *for Cs. annulata* and 100 presence points and 100 absence points for *An. claviger* and *Oc. punctor*.

The maps in Figure 1 show the observed occurrence data (i.e., the model input) and the predicted environmental suitability (i.e., the model outcomes). The ten most important variables for each model and each species are reported in Table 1. Accuracy measures are given in Table 2. For each species, the environmental suitability maps, the most important environmental variables and the model performances are reported below.

**Table 1:** **Most important variables per species and per model**

**SPECIES**	**NLDA**	**RF**	**GLM**
*Cs. annulata*	• Population density	• NLST P2	• EVI VR
	• WORLDCLIM precipitation P2	• Population density	• DEM
	• WORLDCLIM precipitation A0	MIR A2	• DLST A2
	• WORLDCLIM precipitation D1	• DLST A2	• NLST P3
	• WORLDCLIM precipitation DA	• MIR MX	• CMORPH precipitation VR
	• CMORPH precipitation A1	• NDVI A2	• CMORPH precipitation A3
	• DLST DA	• MIR P1	• DLST D1
	• DLST P1	• EVI MN	• DLST D3
	• DLST A0	• CMORPH precipitation P1	• MIR A2
	• DLST P2	• WORLDCLIM precipitation P1	• MIR 03
		• DLST A3	
*An. claviger*	• WORLDCLIM precipitation P2	• NLST MX	• EVI P2
	• WORLDCLIM precipitation A0	• MIR MN	• DEM
	• Population density	• NLST A0	• NLST MN
	• MIR A3	• WORLDCLIM precipitation P3	• NLST A2
	• WORLDCLIM precipitation DA	• NLST MN	• CMORPH precipitation A 1
	• EVI D2	• DLST A0	• MIR D3
	• NLST P3	• DLST A1	• WORLDCLIM precipitation D3
	• EVI P2	• DLST MX	• CMORPH precipitation A2
	• NLST A3	• NDVI A2	• NLST A0
	• DLST A0	• NDVI VR	• Population density
*Oc. punctor*	• Population density	• Population density	• NDVI D1
	• MIR P1	• MIR P1	• MIR P1
	• EVI P3	• EVI P3	• DLST P2
	• NDVI P3	• NDVI P3	• EVI P2
	• NDVI P2	• NDVI P2	• MIR A3
	• DLST MN	• DLST MN	• WORLDCLIM precipitation A3
	• DEM	• DEM	• NDVI A3
	• CMORPH precipitation A2	• CMORPH precipitation A2	• WORLDCLIM P3
	• CMORPH precipitation A1	• CMORPH precipitation A1	• EVI MN
	• WORLDCLIM precipitation P3	• WORLDCLIM precipitation P3	• CMORPH precipitation A2

For non-linear discriminant analysis (NLDA) and generalised linear model (GLM) the top 10 variables average ranks are reported, for random forest (RF) the most important variables are expressed by the mean decrease in Gini index.

**Table 2:** **Accuracy measures for the environmental suitability per species and per model**

**SPECIES**		**NLDA**	**RF**	**GLM**
*Cs. annulata*	Specificity (CI)	0.805 (0.596-0.884)	**0.892** (0.808-0.936)	0.576 (0.442-0.811)
	Sensitivity (CI)	0.639 (0.541-0.829)	0.637 (0.696-0.779)	**0.753** (0.498-0.868)
*An. claviger*	Specificity (CI)	0.670 (0.559-0.850)	**0.908** (0.875-0.944)	0.652 (0.452-0.820)
	Sensitivity (CI)	0.772 (0.567-0.866)	**0.890** (0.827- 0.945)	0.709 (0.512-0.890)
*Oc. punctor*	Specificity (CI)	0.828 (0.735-0.954)	**0.910** (0.825-0.944)	0.765 (0.574-0.828)
	Sensitivity (CI)	0.932 (0.795-1.00)	**0.945** (0.890-1.00)	0.808 (0.685-0.945)

The confidence intervals (CI) are based on 2000 stratified bootstrap replicates.

The best values for sensitivity and specificity for each species are printed in bold.

### Culiseta annulata

*Cs. annulata* has the highest number of observed presences (438, Table 3) in the study. The presence points -indicated by black dots in Figure 1- show that this species was found almost all over the country. The environmental suitability maps show greater suitability in the western part of the Netherlands and lower suitability more inland. Although the areas identified as suitable (and unsuitable) are similar in all maps (Figure 1), the environmental suitability indicator for NLDA has a wider range of values (0.07-0.91) than RF (0.12-0.79) and GLM (0.10-0.86). This is visible in the different intensities of blue and red in the maps. RF and GLM identify also the northern and central part of the country as suitable environment. In all three models, precipitation and land surface temperature are important variables (Table 1). Population density comes up as an important variable in both the NLDA model and the RF model (first and second-most important variable, respectively). Highly forested areas (e.g., National Park Hoge Veluwe situated in the centre of the country) are not identified as suitable for this species in any of the three models. RF is the most specific method (specificity = 0.89, Table 2), while GLM is the most sensitive (sensitivity = 0.75, Table 2).

**Table 3:** **Number of presence and absence points per species**

**SPECIES**	**Presence**	**Absence**
*Cs. annulata*	438	344
*An. claviger*	127	655
*Oc. punctor*	73	709

### Anopheles claviger

The number of presence locations for *An. claviger* was 127 (Table 3). This species does not show a particular pattern in the distribution over the country (black dots in Figure 1). All maps (Figure 1) show lower environmental suitability for this species in the northern and central part of the country whereas the environment is identified as more suitable in the eastern part and in the coastal area (especially for RF and GLM). NLDA predicts much lower suitability values than the other techniques (minimum values: NLDA = 0.07, RF = 0.22, GLM = 0.17), only a few values are larger than 0.5 and as a consequence the average value is very low (average values: NLDA = 0. 27, RF and GLM ≈ 0.50). The GLM map indicates wetlands and floodplains as suitable environments. The most important variables are precipitation, land surface temperature, vegetation indices and middle infra-red, which is a vegetation related index (Table 1). Highly forested areas are not identified as suitable for this species. RF has excellent classification capabilities, the highest when compared to the other techniques (specificity = 0.91, sensitivity = 0.89, Table 2).

### Ochlerotatus punctor

Among the three species presented here, *Oc. punctor* is the least present (73 presence locations, Table 3) and it is the only species showing a clear pattern in the observations (Figure 1); there are more presence points in the east of the country (inland). All three models indicated higher environmental suitability in this part of the country (Figure 1). Comparing the suitability values obtained with the different techniques, the values are higher for RF and GLM and lower for NLDA (maximum values: NLDA = 0.81, RF = 0.94, GLM = 0.92). In the top 10 variables, middle-infra red, vegetation indices, precipitation and day land surface temperature are reported (Table 1). Population density is recorded as the most influential variable in NLDA and RF models. Highly forested areas are identified as suitable for this species in all three models. RF showed excellent classification capabilities (specificity = 0.91, sensitivity = 0.95, Table 2), and it is the modelling technique with the best accuracy.

## Discussion

The environmental suitability for *Cs. annulata*, *An. claviger* and *Oc. punctor* has been investigated using field and environmental data and applying three different modelling approaches, i.e., NLDA, RF and GLM. When comparing the maps for each species produced with the three different modelling techniques, we see consistent spatial patterns, but different levels of predicted environmental suitability. The average predicted environmental suitability was lower for NLDA than for the other methods. This is visible in the predominance of blue colours in the NLDA maps.

Most of the variables highlighted by the models as important are in agreement with field experience, existing biological knowledge, and known habitat preference of these species in Belgium (MODIRISK) [1]. Precipitation and temperature for *Cs. annulata* and *An. claviger* are important in both our study and MODIRISK. For *An. claviger* the population density is also reported as important, both in MODIRISK and in our study (for NLDA and GLM). Both studies show a preference of the latter species for the coastal area. The GLM map for *An. claviger* shows wetland and the floodplains of the big rivers as suitable environments, in accordance with field knowledge. *Oc. punctor* occurrence is related to land surface temperature and particularly with vegetation indices, in the Netherlands as well as in Belgium. This species is generally found in forests and natural areas. Population density is recorded in our study as one of the most influential variables in NLDA and RF models for *Oc. punctor* and *Cs. annulata. Cs. annulata* is known to breed in a wide variety of habitats and to be associated with areas with human activity [5], whereas *Oc. punctor* prefers swampy forest with boggy waters and seldom flies out of the forest [5], characteristics that suggest a negative relationship with human presence.

In terms of model performance, RF shows the best discrimination skills. Also in other studies, this technique was consistently reported to outperform other traditional modelling techniques [6,7]. Only the GLM for *Cs. annulata* has a higher sensitivity than RF. Random forest sensitivity and specificity are excellent, often equal to or larger than 0.9. However, if we want to interpret these values, we have to consider that the training data are also used to evaluate the model, meaning that the accuracy measures will be overestimated [8,9]. Although most modellers consider that external validation is preferable to internal, there are cases where internal validation (i.e., the model ability to fit the training data) is sufficient. If the goal is to describe a pattern, overestimating the accuracy is not a problem. This is the case for models seeking to convert the observed records of a species into a suitability score [10], as in our study. Overall, when making predictions based on occurrence data, presence data are more reliable than absence data [11]. Absence points may represent areas where the trap failed to catch a mosquito despite these mosquitoes being present in the area, or areas that are in principle suitable, but which have not yet been invaded. Therefore, in these cases, it is recommended to prioritize the sensitivity over the specificity [12].

Sensitivity and specificity measures were used to compare techniques for the same species and in the same geographical area. It was not possible to compare model performance between species because the traditional methods are highly influenced by the relative areas of occurrence of different species and by the geographical extent: increasing the geographical extent outside the presence environmental domain leads to a larger score for the area under the curve [13]. In fact, it has been shown that the relative occurrence area of the species influences the results of the evaluation scores, implying that models of rare species with high environmental specificity will yield to higher discrimination values [14,15] and that species with restricted environmental tolerance and/or distributions are usually reported to be well predicted [16]. This is indeed what we would observe if we compared sensitivity and specificity between species: *Oc. punctor* is mainly observed in the east of the country and the random forest has the highest discrimination skills, compared to other species.

To create a reliable model, it is generally considered necessary to have the same number of presence and absence points as input. This is because having a different number will create a bias in the model prediction towards the more prevalent category (presence or absence) [17]. For NLDA and GLM, this balancing, i.e., considering the same number of presences and absences, is accomplished at the bootstrapping stage. However, for techniques such as random forest it is necessary to select a ‘balanced’ subset of the data.

Some areas were excluded from the sampling scheme, because they were deemed unsuitable for mosquitoes. At the modelling stage, this had to be corrected by adding absence points in these unsampled regions (for details, see methods section). In our study, this adjustment was possible because it was known that the unsampled regions would be negative for presence of mosquitoes. Generally speaking, avoiding bias in sampling strategies is more advisable than correcting for lack of data at the modelling stage.

The aim of the study was to investigate the spatial distribution of mosquito species and to compare the performance of three statistical models. In recent years, predictive modelling of species distribution has become an increasingly important tool to address various issues in ecology, biogeography, evolution, conservation biology and climate change research [18]. Beyond describing species distributions, these models have become an important and widely used decision making tool for a variety of biogeographical applications, such as mapping risk of vector-borne disease spread, and determining locations that are potentially susceptible to invasion [19]. Species distribution modelling using flexible machine learning approaches has been successfully applied to quantify and to map the global distribution of hosts [20], disease vectors [21], pathogens [22], and infection and outbreak risk [23].

## Conclusions

In this study we mapped the environmental suitability for three mosquito species with three different modelling techniques. For each species, the models produced consistent spatial patterns, but different levels of prediction ranges. The average predicted environmental suitability was lower for NLDA than for the other methods. The variables selected as important in the models were consistent with field experience and the existing knowledge about these species. All the modelling techniques showed a satisfactory to excellent accuracy; the best accuracy was obtained with the random forest model.

The insights obtained in this study can be used to improve future predictions for vector and non-vector species. The output of this type of distribution modelling methods can be used as input for epidemiological models and can be helpful to identify suitable areas for a given species, at risk of successful invasion if the species is still absent. Such areas may therefore need particular attention in terms of measures of prevention.

## Methods

The input for spatial distribution modelling consists of mosquito field data and environmental, often satellite, data. Here we describe the mosquito data collection, the environmental data used and the statistical methods applied.

### Mosquito data

Data were collected by the Dutch Centre for Monitoring of Vectors during the National Mosquito Survey program, from April to October 2010–2013 [24]. These consisted of mosquito abundance data, sampled at 778 locations. Each of the locations was sampled only once and each trap was active for one week. At the sampled locations, mosquitoes were captured by means of CO_2_-baited Mosquito Magnet Liberty Plus MM3100 traps (Woodstream® Co., Lititz, USA). These traps have been evaluated successfully for trapping and surveillance against a variety of mosquito genus and species [25,26], and have been used successfully in the national inventory of mosquitoes in Belgium, MODIRISK [2], also to capture *Culiseta annulata*, *Anopheles claviger* and *Ochlerotatus punctor.* For our survey, the traps were randomly located in the Netherlands, following the study design described in Ibañez-Justicia et al. [24]. Of the traps, 40% were placed in urban areas, 40% in agricultural areas and 20% in natural areas. Natural areas were sampled to a lesser extent because of their presumed lower involvement in human and veterinary health risks.

**Figure 2:**
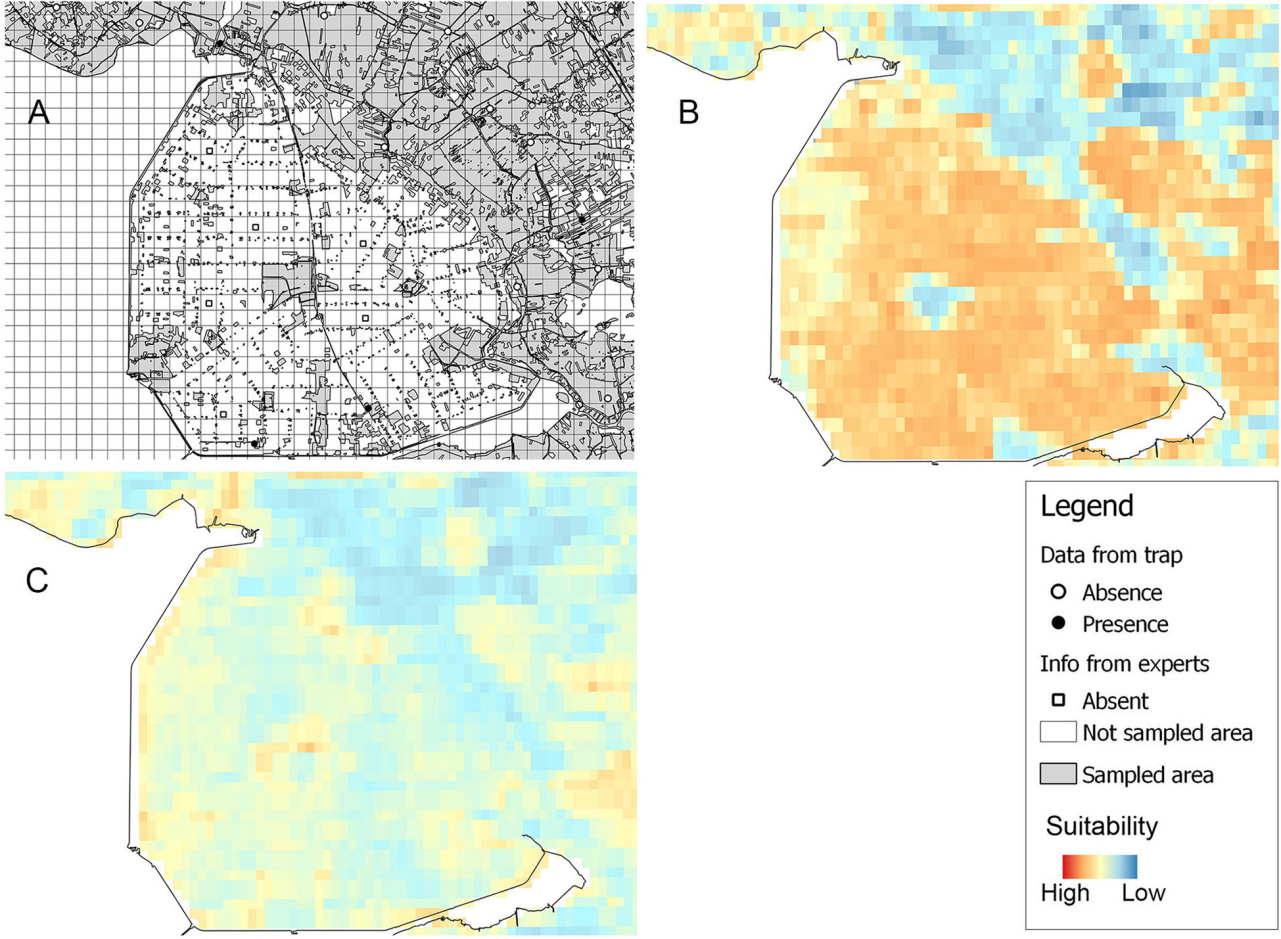
**Probable absences added in unsuitable unsampled areas. A** – Absence points added to the *An. claviger* data in part of Flevoland province. The grey area was sampled and the white area was excluded from the sampling because it was considered unsuitable for mosquitoes. White and black circles indicate negative and positive traps, respectively. White squares indicate the probable absences added in unsuitable unsampled areas. **B** – Random forest predictions for *An. claviger* without pseudo-absences. Environmental suitability is depicted using a gradient fill: blue indicates low environmental suitability, red indicates high suitability. **C** – Random forest predictions for *An. claviger* with pseudo-absences.

As described in Ibáñez-Justicia et al. [24], high productivity agricultural areas, such as arable land or permanent crops, were not sampled as they are considered to be unsuitable due to a lack of mosquito breeding sites. Therefore, areas with beet, grain, maize, potatoes and other agricultural crops, bulb flowers, productivity orchards and greenhouses were excluded from the sampling. When the goal is to estimate the potential distribution of a species, it is important that absence data come from environmental conditions that are known to be unsuitable for the species [27]. If information on absence is not available, absences can be generated outside the environmental domain where the species is present [14]. This has been done, for example, in Jiménez-Valverde and Lobo [28], where probable absences were randomly selected in the areas having environmental values outside the range of observed presences. In a similar way, forty-three absence points in our study were generated in the land cover types that are deemed unsuitable for mosquitoes. Omitting this information, would have led to unrealistic predictions, as is for example shown in Figure 2. In Figure 2A, the presence and absence points for *An. claviger* are shown as black and white circles in an area of Flevoland province (north-west). The grey area was sampled and the white area was excluded from the sampling because it was considered unsuitable for mosquitoes. Only two traps were located in this part of the region, in two fragments that were part of the sampled area, and they were both positive for the presence of mosquitoes. Both positive traps were located in small areas enclaved in pixels that were mainly considered to be unsuitable (intensively used agricultural fields). However, these possibly unsuitable areas were not sampled and therefore there are no data informing the model of their unsuitability. In a model without probable absence added, the two pixels are identified as suitable and consequently the whole area will be predicted as being suitable (Figure 2B), potentially incorrectly. If we introduce also absence points, the model is provided with more complete information and gives more realistic predictions (Figure 2C).

The abundance data were reclassified into data of presence (when at least one mosquito was found in the trap) and absence (when no mosquitoes were found in the trap), because the number of mosquitoes in each location was measured in different weeks and the mosquito abundance is expected to vary seasonally. The resolution used for the maps is 1 km^2^. When a presence and an absence point were in the same square kilometre only the presence point was selected because presences inform about the places that are environmentally suitable for a species, but absences do not necessarily indicate the opposite [29]. This reduced the number of locations used in the analysis from 778 to 766.

### Environmental variables

The environmental data included in the analysis as predictor variables are 1 km^2^ resolution satellite images and meteorological data in raster file format, commonly used for mosquito distribution modelling [30]. The images were obtained from the MODIS sensor on NASA’s Terra and Aqua satellites [31,32] for 2000–2012 and subjected to temporal Fourier transformation [33,34] to summarise the images and to produce sets of data that capture characteristics of the annual seasonality: the mean, the annual bi-annual and tri-annual amplitudes and phases, the maxima, minima and variances of variances of the middle infra-red (MIR), the daytime Land Surface Temperature (dLST), the night-time Land Surface Temperature (nLST), the Enhanced Vegetation Index (EVI) and the Normalized Difference Vegetation Index (NDVI) signals [35]. Other environmental data used in this study are precipitation (WorldClim [36] and CMORPH [37] 1950–2000), population density (compiled from the Gridded Population of the World Dataset 2000 [38]), the digital elevation model (MODIS [32] 2012) and land cover (Corine land cover map of 2006) [39]. A list of the Fourier components is provided in Table 4 and the environmental data are listed in Table 5. Predictor variables were organized as raster type files and for each trap location the pixel values of the environmental variables were extracted.

**Table 4:** **Fourier components from temporal Fourier analysis of an imagery time series**

**Component**	**Description**
A0	Fourier mean for entire time series
MN	Minimum value
MX	Maximum value
A1	Amplitude of annual cycle
A2	Amplitude of bi-annual cycle
A3	Amplitude of tri-annual cycle
VR	Total variance
P1	Phase of annual cycle
P2	Phase of bi-annual cycle
P3	Phase of tri-annual cycle
D1	Proportion of total variance due to annual cycle
D2	Proportion of total variance due to bi-annual cycle
D3	Proportion of total variance due to tri-annual and cycle
DA	Proportion of total variance due to all three cycles

Component is the name used in the software Vecmap.

**Table 5:** **Environmental predictor variables**

**Source**	**Variable**
MODIS	Middle infra-red (MIR)
MODIS	Day-time land surface temperature (DLST)
MODIS	Night-time land surface temperature (NLST)
MODIS	Enhanced vegetation index (EVI)
MODIS	Normalised difference vegetation index (NDVI)
CMORPH	Precipitation
WorldClim	Precipitation
MODIS	Digital elevation model (DEM)
Gridded population of the world	Human population density
European Environment Agency	Corine land cover

### Statistical analysis

Species distribution models quantitatively describe areas that support the presence of a given species, based on known occurrence data and the associated environmental conditions [40]. Here, three methods suitable for occurrence data have been applied, i.e., non-linear discriminant analysis, random forest analysis and a generalised linear model, aimed at describing the relationship between response and predictor variables. For all three modelling techniques, the output was an environmental suitability indicator for each species, expressed as a value between 0 (low suitability) and 1 (high suitability). The predicted environmental suitability is visualised in maps.

#### Non-linear discriminant analysis

Models created using NLDA [34] require presence and absence data to be grouped into clusters based on attribute data. In this way, a discriminant function can be created and predictive maps based on these clusters can be made. The main advantage of the clustering is that it handles spatial heterogeneity of habitat niches and zones. The data were clustered using the k-means clustering algorithm. Since the important variables for the species were unknown, generic variables were used for clustering. These variables were DEM and the means, amplitudes, maxima, minima, variances (of the entire signal) and phases of MIR, LST and NDVI. NLDA models were bootstrapped [41], meaning that 100 models were run and that for each model a sample of an equal number of presence and absence points was taken from the training set with replacement. The final predictions are based on the average of the 100 models.

#### Random forest

A random forest [42] method consists of an ensemble of classification and regression trees (CART; [43]) constructed using a random subset of both the available samples and the attributes recorded for each data point. A CART tree is a hierarchical structure that allows a data point to be assigned to a particular class based on its attribute values. For the random forest method it is necessary to have the same number of presence and absence points as input, in order to obtain unbiased model predictions [44]. For techniques such as NLDA and GLM, this balancing is guaranteed at the bootstrapping stage, but this is not the case for RF. Therefore, before the model was run, five ‘balanced’ subsets of the complete dataset were randomly created. For each species, if there were more absence than presence points, all the presence points were used and a random subset of the absences was selected. If the presence points outnumbered the absence points, the procedure was inversed. The RF models were not bootstrapped because inherently RF uses a rationale similar to the bootstrapping approach, being based on several CART trees. The final predictions are the average of the five submodels.

#### Logistic regression

For the GLM analysis, a logistic regression model was used because the response is a binary variable (presence/absence). GLM models can account for spatial autocorrelation by using an autoregressive term or mixture model. The effect of spatial autocorrelation on the training set was checked in the correlograms, i.e., plots of distance between points and the Moran’s I index of their correlation. Since the correlation effect was not strong (with the exception at extreme distances where it is known that values for Moran’s I may be erratic due to fewer points that can be compared [45]) there was no need to account for spatial autocorrelation. As for NLDA, the GLM models were bootstrapped 100 times with a sample of an equal number of presence and absence points after which the 100 models were averaged to produce the final predictions.

#### Model evaluation

The choice of evaluation strategy needs to be explicitly related to the subject and goals of modelling. Here, the aim is to describe a given pattern and get a suitability score. In this context, simple forms of verifications, e.g. the number of false negatives, is appropriate to check whether models are performing as intended [10]. For each model, sensitivity and specificity were calculated, where sensitivity is the ability of a model to correctly identify known positive sites and specificity is the ability of a model to correctly identify known negative sites. Sensitivity and specificity were reported together with the values of their confidence intervals. The confidence intervals are calculated based on 2000 stratified bootstrap replicates at 95% level. Sensitivity and specificity were used to compare the results produced with NLDA, RF and GLM for the same species. A list of the most important variables used in the models is provided. For NLDA and GLM the top 10 ranked variables are listed and for RF variable importance is given as mean decrease in Gini index [42,43].

The analysis has been performed with the software Vecmap demo version [46]. The accuracy measures have been calculated with R 3.0.2 statistical language environment [47] (R Development Core Team 2013), using of the R-packages pROC, ROCR, OptimalCutpoints.

## Abbreviation

NLDA: Non-linear discriminant analysis
RF: Random forest
GLM: Generalised linear model
*Cs.*: *Culiseta*
*An.*: *Anopheles*
*Oc.*: *Ochlerotatus*
MIR: Middle infra-red
dLST: Daytime Land Surface Temperature
nLST: Night-time Land Surface Temperature
EVI: Enhanced Vegetation Index
NDVI: Normalized Difference Vegetation Index
DEM: Digital Elevation Model
